# Vaccination Promotion Strategies in the Elderly: Systematic Review and Meta-Analysis

**DOI:** 10.3390/vaccines12121395

**Published:** 2024-12-11

**Authors:** Ana Pereira, Cláudia Pinho, Adriana Oliveira, Rui Santos, Miguel Felgueiras, João P. Martins

**Affiliations:** 1 ESS, Polytechnic of Porto, Rua Dr. António Bernardino de Almeida 400, 4200-072 Porto, Portugal; 10220113@ess.ipp.pt (A.P.); clp@ess.ipp.pt (C.P.); 2 REQUIMTE/LAQV, ESS, Polytechnic of Porto, Rua Dr. António Bernardino de Almeida 400, 4200-072 Porto, Portugal; 3 Centre for Organisational and Social Studies of the Polytechnic Institute of Porto (CEOS.PP), Accounting and Business School (ISCAP), Porto Polytechnic Institute, Porto, 4465-004 São Mamede de Infesta, Portugal; aoliveira@iscap.ipp.pt; 4 Escola Superior de Tecnologia e Gestão, Instituto Politécnico de Leiria, Campus 2, Morro do Lena—Alto do Vieiro, Apartado 4163, 2411-901 Leiria, Portugal; rui.santos@ipleiria.pt (R.S.); mfelg@ipleiria.pt (M.F.); 5 CEAUL—Centro de Estatística e Aplicações, Faculdade de Ciências, Universidade de Lisboa, 1150-082 Lisboa, Portugal

**Keywords:** elderly immunization, intervention, strategies, vaccination promotion

## Abstract

**Background:** The World Health Organization estimates that currently available vaccines prevent 2 to 3 million deaths worldwide each year. Preventing infectious diseases is an important public health priority to ensure healthy ageing and improve quality of life. This study’s aim is to identify the best strategies to increase vaccination coverage in the elderly. **Methods**: A systematic review and meta-analysis were carried out, including a bibliographic search in the PubMed and Scopus databases. Studies in older people (60 years or older) on any type of intervention aimed at increasing vaccination coverage were included. The effect of the intervention was measured using the odds ratio (OR). **Results**: After applying the selection criteria, 20 studies were identified: 17 on influenza vaccines and 3 on other vaccines. Educational strategies obtained an OR = 1.63 (95% CI: 1.22–2.19, $I^{2}$ = 0.59). Medical counselling obtained an OR = 3.13 (95% CI: 0.60–16.37, $I^{2}$ = 0.95). Writing strategies obtained an OR = 1.14 (95% CI: 0.99–1.32, $I^{2}$ = 0.93). Few studies reported the effect of free vaccination. **Conclusions**: The educational strategies proved to be more effective than the others in this study. Free vaccination and age may have important roles. Further studies are needed as research in this area remains limited.

## 1. Introduction

The World Health Organization (WHO) estimates that the current vaccine programme prevents 2 to 3 million deaths worldwide each year. The WHO considers vaccines to be the most effective and cost-effective medical intervention for the prevention of deaths and the reduction of illness in communities [1,2,3]. Vaccines are regarded as the primary tools for preventing human infection, representing one of the most significant public health advancements of the past century. They are effectively employed in combating a range of bacterial and viral infectious diseases [2,3].

Vaccines are composed of inactivated or attenuated viruses that elicit an immune response in the host without causing disease. Upon administration of a vaccine, the immune system responds to the introduced pathogens by generating antibodies that can protect against future infections [4]. It is therefore evident that vaccination is of significant importance, as it offers protection against a specific infectious disease, while also safeguarding against potential future complications that may arise as a result of this disease, whether immediate or long-term [3,4]. While a number of vaccines offer lifelong immunity, some necessitate a booster dose due to the attenuation of their protective effects with age [2]. The administration of the vaccine results in immunization, which is designed to reduce the prevalence and ramifications of catastrophic infections [1]. The greater the proportion of the population that has been vaccinated against a given disease, the lower the likelihood of its occurrence, thereby affording protection to those most vulnerable to its effects, such as the elderly [4].

The immune system undergoes age-related changes, known as immunosenescence, which increase the incidence and severity of infections. These changes also reduce vaccine responsiveness in older adults. With rising life expectancy, the global population is ageing, and the number of individuals over 60 is projected to double by 2050, reaching 2.1 billion. This demographic shift presents significant challenges for public healthcare systems, as older individuals are more susceptible to frequent and severe infections than younger populations [5].

Preventing infectious diseases is a critical public health objective to promote healthy ageing and enhance quality of life. Adhering to recommended vaccination schedules is an effective strategy to mitigate the impact of these diseases in the elderly. The ageing population underscores the need for public health policies and pharmaceutical innovations tailored to this demographic [6].

In many countries, such as the United States, European Union member states, and China, vaccines against influenza and Streptococcus pneumoniae are recommended for elderly individuals, as these pathogens are major causes of severe morbidity and mortality in this age group [7]. Vaccination against diphtheria and tetanus is also part of the general immunization schedule and is particularly emphasized for elderly individuals. More recently, the Herpes Zoster (HZ) vaccine has been recommended to address the rising incidence and severity of HZ and post-herpetic neuralgia [6].

With the emergence of SARS-CoV-2, the COVID-19 vaccine is now recommended for older adults due to the high mortality rates associated with the virus in this population [8].

High vaccination coverage prevents outbreaks and controls seasonal endemic outbreaks, thereby promoting a resilient society [9,10]. However, vaccination rates do not always reach the desired levels [7]. Exposure to misinformation can increase vaccine hesitancy [11]. While mandates and restrictions based on vaccination status may boost vaccination rates, they can also lead to counterproductive outcomes, which should be carefully considered [12].

It is thus imperative to devise and execute strategies and campaigns that foster awareness of the significance of vaccination and enhance compliance with vaccination protocols to prevent the occurrence of diseases within the general population and, in particular, within the risk groups identified for each disease [13].

The main objective of this systematic review was to identify which interventions are most effective in increasing adherence to vaccination in the elderly.

## 2. Materials and Methods

The methodology proposed for this systematic review was registered in the International Prospective Register of Systematic Reviews with PROSPERO registration number CRD42023426119. This review followed the criteria of the Preferred Reporting Items for Systematic Reviews and Meta-Analyses (PRISMA) [14].

### 2.1. Research Strategy

The literature search was conducted in MEDLINE (via PubMed) and Scopus databases. The search strategy was defined using specific database vocabularies.

The bibliographic search in PubMed was performed using the following search terms:

#1(vaccine[MeSH Terms]) AND (hesitancy[TIAB]) AND (intervention [TIAB] OR knowledge [TIAB] OR attitudes [TIAB] OR behaviour [TIAB] OR immunization programs [TIAB] OR strategies [TIAB] OR elderly [TIAB]);

#2(vaccine[MeSH Terms]) AND (uptake[TIAB]) AND (intervention [TIAB] OR knowledge [TIAB] OR attitudes [TIAB] OR behaviour [TIAB] OR immunization programs [TIAB] OR strategies [TIAB] OR elderly [TIAB]).

The bibliographic search in Scopus was carried out using the following search terms: (vaccine) AND (hesitancy) AND (intervention OR knowledge OR attitudes OR behaviour OR immunization programs OR strategies OR elderly) in Title Abstract Keyword OR (vaccine) AND (uptake) AND (intervention OR knowledge OR attitudes OR behaviour OR immunization programs OR strategies OR elderly) in Title Abstract Keyword (word variations have been searched).

### 2.2. Condition or Study Domain

The scope of the study was any type of intervention designed to increase immunization coverage for any WHO-approved vaccine. Studies that only assessed changes in intention to vaccinate were excluded.

### 2.3. Participants

This review only included trials of older people (people aged 60 or over). Trials were included if they included other age groups but reported separate results for groups of people tested for this condition.

### 2.4. Intervention

Any planned intervention to increase vaccination coverage was included. These interventions may include, for example, communication, education and information strategies using any means (e.g., letters, leaflets, educational campaigns, media and social media campaigns, vaccination campaigns), as well as reminders, calls to action and incentives, and interventions to increase access through different delivery models, such as clinics and home visits. Provider or system-based interventions (e.g., provider evaluation and feedback, provider incentives) were excluded.

### 2.5. Comparator

The comparator is no intervention, i.e., usual care. Trials that only compared interventions were not included.

### 2.6. Types of Studies

Controlled comparative intervention studies were included, including randomised clinical trials, quasi-experimental studies, and observational studies, including cohort and cross-sectional studies.

### 2.7. Inclusion and Exclusion Criteria

Studies were included that quantitatively assessed the effects of strategies and interventions on vaccine uptake in older people, which is an indicator of vaccine adherence, including single vaccinations and/or completion of a complete vaccination documented in medical records. Only articles published between 2016 and 2023 and in Portuguese or English were included.

Studies were excluded if they reported on issues of vaccine adherence, but did not describe specific interventions used to influence vaccine adherence and did not provide a quantified assessment of vaccine adherence. Self-reports were also excluded due to the possibility of memory decline, which is often present in this population [15].

### 2.8. Measures of Effect

The desired outcome was vaccination uptake. The effect of the intervention was measured using an odds ratio (OR). If the OR equals one, it means that the intervention had no effect. A value greater than 1 means that the intervention was effective, and a value less than 1 means that the intervention reduced the vaccination rate.

When studies reported both unadjusted and adjusted ORs, the adjusted figure was used in the results as it was considered the less biased estimate of the intervention effect.

Additional outcomes of interest were collected for subgroup analysis.

### 2.9. Data Extraction (Selection and Coding)

Study eligibility was assessed independently by two authors by analysing the title and abstract of the articles. Following this selection, the full article was also read by two authors to assess whether it met the inclusion criteria. Information from the same articles was independently extracted into a form that includes the study design, sample size, description of intervention, outcomes, and quality indicators. Disagreements about the selection of studies were resolved by consensus.

### 2.10. Risk of Bias Assessment

The risk of bias in the included studies was assessed independently by two authors using validated critical appraisal tools. Disagreements were resolved by consensus.

The Newcastle–Ottawa tool (NOS) was adapted to assess the methodological quality of the studies included in this meta-analysis, considering the specificities of this study (see Supplementary Table S1). This tool considers the following areas of evaluation: the selection of participants (scored from 0 to 4), the comparability of results, namely the control of confounding factors (scored from 0 to 2), and the way in which the variables of interest were evaluated (scored from 0 to 3). The total score can vary from 0 to 9. A higher score indicates a higher-quality study. Studies scoring at least 8 points were considered to be of high quality [16].

For the clinical trials, we used the Cochrane Risk of Bias in Randomised Trials tool (RoB 2). It is divided into five areas of potential bias: the randomisation process (D1), the intervention (D2), the potential lack of outcome data (D3), the assessment of outcomes (D4), and the formalisation of outcomes (D5). The overall risk of bias is then classified into 3 categories: low risk of bias (+), some concerns (!), or high risk of bias (−). If studies at high risk of bias were identified, a sensitivity analysis was performed. [17].

### 2.11. Data Synthesis Strategy

Following the systematic review, a meta-analysis was conducted using the R software package meLtafor [18]. The confidence intervals associated with the effect measures were based on log transformation. Heterogeneity between studies was assessed using the $I^{2}$ statistic: a value greater than 0.75 was considered to indicate high heterogeneity, and the corresponding estimates were interpreted as evidence of low quality; if less than 0.25, a fixed effects model was fitted; if greater than 0.25 and less than 0.75, a random effects model was fitted [19]. The models were estimated using restrictive maximum likelihood estimation with the Knapp and Hartung adjustment.

Funnel plots were presented for visual assessment of possible publication bias. The trim and fill method and Egger’s test were used to analyse publication bias [20]. If missing studies were identified by the trim and fill method, the pooled effect estimates were recalculated after imputing these missing studies to provide adjusted estimates that account for potential bias.

### 2.12. Subgroup or Subset Analysis

To analyse the sources of heterogeneity in the studies (with the aim of reducing this heterogeneity), a subgroup analysis was conducted considering the type of intervention, the Human Development Index (HDI) of the country where the study was carried out (classified into four categories as proposed by the United Nations Development Programme in 2024: very high, high, medium, and low HDIs [21]), as well as the minimum and mean age and sex of the participants. The comparison of ORs between subgroups was performed using Cochran’s Q test. Only subgroups with at least 4 trials were considered.

## 3. Results

### 3.1. Selection of the Studies

**Figure 1:**
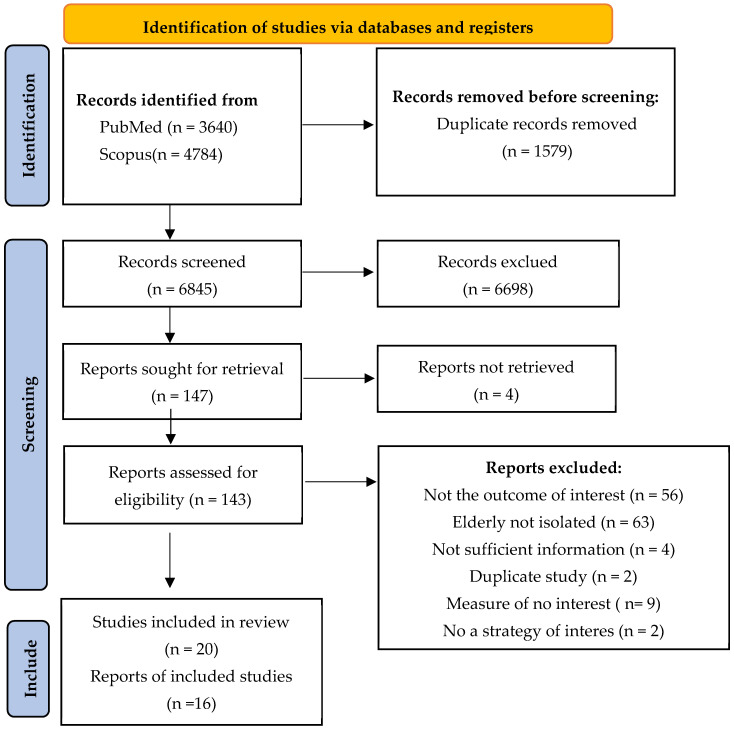
PRISMA study search flow diagram.

The search yielded 3640 articles from the PubMed database and 4784 articles from the Scopus database, resulting in a total of 8424 articles on vaccination strategies that were included in this systematic review. A total of 1579 duplicate articles were identified in both databases, and thus excluded from the subsequent analysis. This resulted in a total of 6845 articles requiring analysis. Following a review of the titles and abstracts, 6698 articles were excluded. Following the application of the inclusion and exclusion criteria, a total of 16 articles and 20 studies on interventions to promote vaccination in the elderly were included in this systematic review, as illustrated in Figure 1.

The strategies with the most studies are free vaccines [22,23,24], SMS reminders [25,26,27], and educational letters [28,29,30]. Additionally, the strategies include, though with fewer studies, emails [29,31], medical recommendations [15,32], individualised advice [33,34], information leaflets [35], educational videos [36], through television [15], and through community councils [15].

The study population included individuals aged 60 years or older (8 studies) and 65 years or older (12 studies). All studies were conducted in countries classified as having a high (8 studies) or very high (12 studies) HDI. Information on the age and sex of participants was available in only 8 and 13 studies, respectively, as most studies reported data for the entire sample, which often included a broader population beyond just the elderly. In some cases, the population exhibited specific comorbidities [25,31], while in others, participants were affiliated with a particular local health centre [23,35]. The sample size varies from 150 people [24] to 384,913 people [31]. The most common type of study in the articles are randomised clinical trials [26,27,29,30,31,33,34,36], then quasi-experimental studies [22,23,24,25]. Most of the vaccines in the studies are against the influenza virus [15,22,23,24,25,26,27,30,31,32,33,34,35,36], as well as, but to a lesser extent, the Pneumococcal vaccine [1;11], and finally against the COVID-19 virus [29].

The OR ranged from 0.9546 (95% CI 0.9075–1.0041) to 30.46 (95% CI 20.86–44.49). Among these values, 11 studies present adjusted OR estimates [15,23,24,26,28,33,35,36]. Of the 20 studies included in this review, only 5 reported a non-significant effect [15,27,30,33,35]. The remaining studies described a positive effect of the intervention under study. Further details of the included studies can be found in Table 1.

### 3.2. Quality of the Studies Included

**Figure 2:**
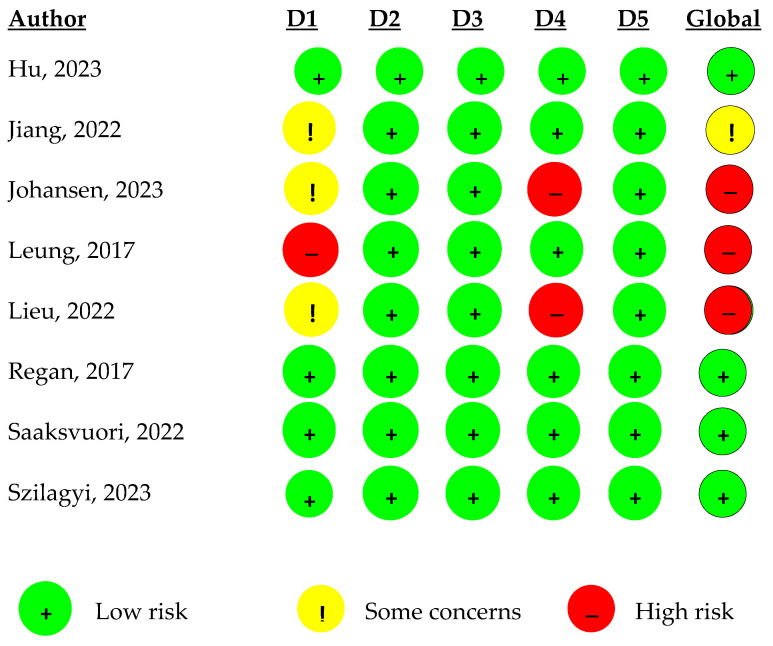
Quality of clinical trial studies (D1: randomisation process; D2: intervention process; D3: lack of outcome data; D4: outcome assessment; D5: formalisation of results) [26,27,29,30,31,33,36,37].

The risk of bias in clinical trial studies was assessed using the RoB 2 tool. It was found that in domains D2, D3, and D5, all the articles presented a low risk of bias. In domain D4, there was a high risk of bias in two articles [29,31]. In domain D1, only one study presents a high risk of bias [34], as described in Figure 2.

Overall, half of the clinical trial studies present were found to have intermediate and high risks of bias, with only four studies having a low risk of bias.

In clinical trials, the randomisation process is an important factor in preventing bias. Blinding of both the participants and the evaluators is essential to ensure that there is no bias in the final result. In some articles, the fact that this concealment is not described [29,31,36] or does not exist [34] creates a high risk of bias and affects the overall risk of bias assessment. The selection of participants should be representative. When a selection was made in one city only, this has been shown to bias the sample towards those who were willing to participate and receive the vaccine, thus limiting the generalisability of the results [36]. A short follow-up period may underestimate long-term effects [34,36], so future studies should be longer. And finally, the control and intervention groups should receive similar treatment to ensure that any differences in outcomes are the result of the intervention and not an effect of the intervention [34].

In the observational studies, the risk of bias was assessed using the NOS. Results are presented in Table 2.

Out of a total of eight articles, only four had a score of eight or more [24,25,32,35]. When analysing by rating domains, comparability of results was the only domain that received the maximum score (two stars) in all articles, thus providing greater control over the confounding factors of the study. In the area of participant selection, only two articles received four stars [24,25]. Finally, in the exposure item, only four articles received three stars [24,25,32,35].

In observational studies, there were some shortcomings in the quality assessment. Failure to mention how the control group was selected or how exposure was determined is often found in articles with a higher risk of bias [15,22,23,28]. Selection bias is introduced by recruiting people who are more likely to be health literate and more accepting of vaccination than the general population [22]. Another reason is that the selection was only made in one city, which does not guarantee that the whole population is representative, and future studies will need to include more randomly selected samples in different locations to provide strong evidence of representativeness [23,28]. The fact that the data were collected at a single point in time, without follow-up, limits their causality, and longitudinal evaluation is needed in the future [22]. It is also possible that memory bias may be present in these studies, as there are articles that employed questionnaires to investigate knowledge about vaccine uptake. The data are limited due to the potential impact of age-related memory decline on the ability to recall information and perform tasks that require specific cognitive abilities [15,32].

### 3.3. Meta-Analysis

The overall estimate of all the interventions included in this systematic review was OR = 2.193 (95% CI: 1.43–3.361, $I^{2}:99.50\%$, *p* < 0.001). The high heterogeneity emphasizes the need to analyse the different strategies involved.

Thus, it was essential to categorize the diverse strategies identified in the studies. The aforementioned categories comprise educational sessions, which encompass strategies conveyed through informational leaflets, posters, and videos, and the promotion of these sessions through television and community councils. The term “medical advice” encompasses recommendations from medical professionals and individualised guidance on the significance of immunization. Free and subsidised vaccinations are another category, and, finally, another is writing strategies such as letters, emails, and SMS messages. Following this categorization, an analysis was conducted to determine which group of strategies was most effective in ensuring adherence to vaccination among the elderly, both individually and comparatively.

At the end of this section, a comparison of participant characteristics—including age, sex, and the country in which each study was conducted—is presented. A comparison between rural and urban samples was not conducted, as only one study was carried out in a rural setting.

#### 3.3.1. Educational Sessions

**Figure 3:**
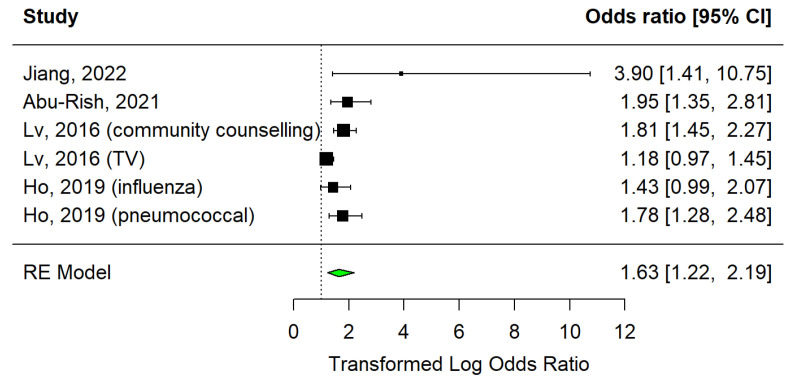
Forest plot of educational strategies [15,28,35,36].

In the educational sessions category, there were four articles that presented information leaflets [35], educational letters [28], videos [36], promotion through television [15], and community councils [15] as strategies under study. Results are displayed in Figure 3, showing six studies, in which only two did not indicate a significant effect [11;21]. Overall, this strategy has a positive and significant effect on increasing vaccination (OR = 1.63, 95% CI: 1.22–2.19, $I^{2}$ = 0.59).

**Figure 4:**
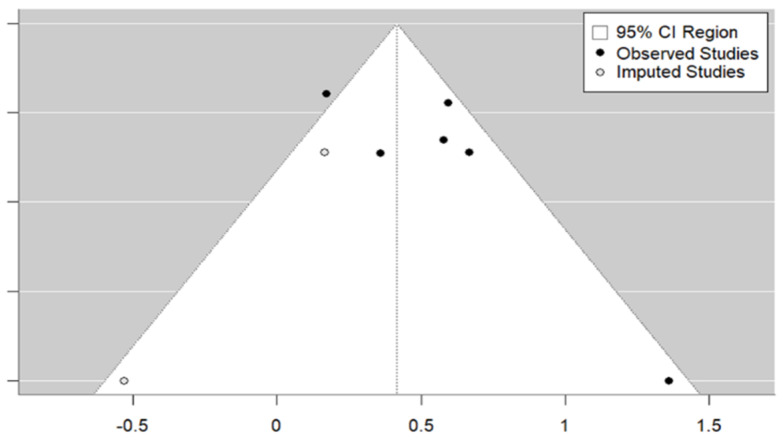
Educational strategy funnel plot.

With regard to the subject of educational strategy, Figure 4 demonstrates that the sample of studies is representative and that there is symmetry and homogeneity across the studies. Egger’s test revealed the absence of publication bias (*p* = 0.0979). The trim and fill method identified two studies that were not included in the analysis. The overall estimate decreases to 1.52 (95% CI: 1.25–1.84) when the studies imputed by the trim and fill method are included.

#### 3.3.2. Medical Advice

**Figure 5:**
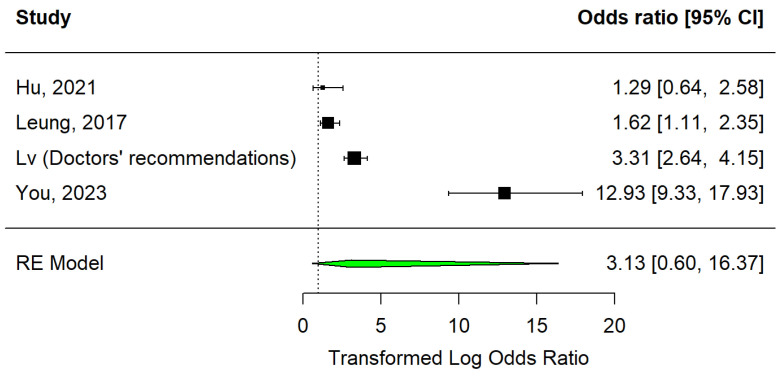
Forest plot of medical counselling strategy [29,32,33].

In the medical counselling category, there were four articles that presented recommendations [15,32] and individualised counselling [33,34] as strategies under study. Data are represented in a forest plot (Figure 5). It shows that out of the four studies, only one showed a non-significant effect [33]. Overall, this strategy has not significantly increased vaccination (OR = 3.13, 95% CI: 0.60–16.37, $I^{2}$ = 0.95).

**Figure 6:**
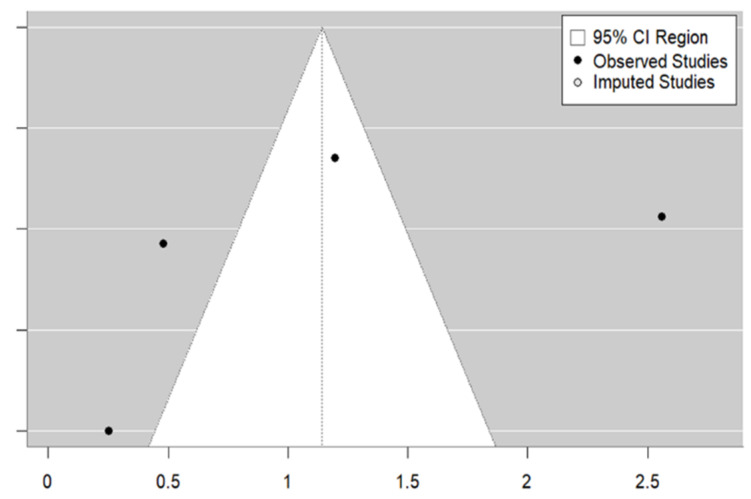
Medical counselling strategy funnel plot.

Still on the subject of medical counselling, and despite the small number of studies, Egger’s test indicates the absence of publication bias (*p* = 0.4448). The trim and fill method did not identify any missing studies (see Figure 6).

#### 3.3.3. Free and Subsidised Vaccination

A meta-analysis was not feasible as the number of available studies did not meet the predefined criteria. Only three articles with this intervention were included in this review [22,23,24]. However, it is worth noting that the reported odds ratios (ORs) range from 5 to 30.46, all of which are among the top four OR values.

#### 3.3.4. Writing Strategy

**Figure 7:**
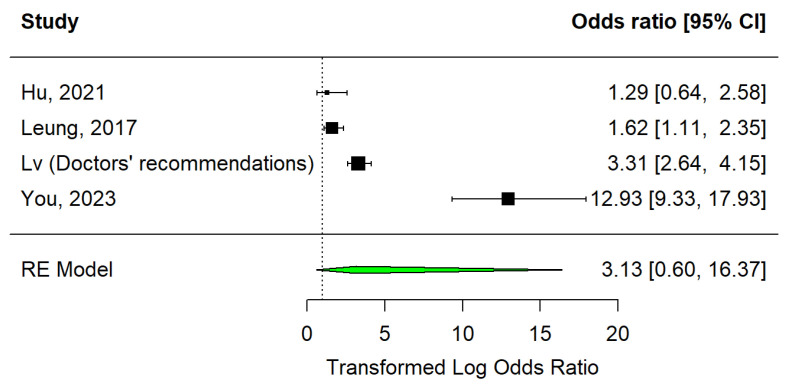
Forest plot of writing strategies [29,32,33].

In the writing strategy category, there were six articles that presented letters [29,30,31], emails [29], and SMS messages [25,26,27] as strategies under study. Figure 7 shows a forest plot of the seven studies, in which only two did not show a significant effect [27,30]. Overall, this strategy has not significantly increased vaccination (OR = 1.14, 95% CI: 0.99–1.32, $I^{2}$ = 0.93).

**Figure 8:**
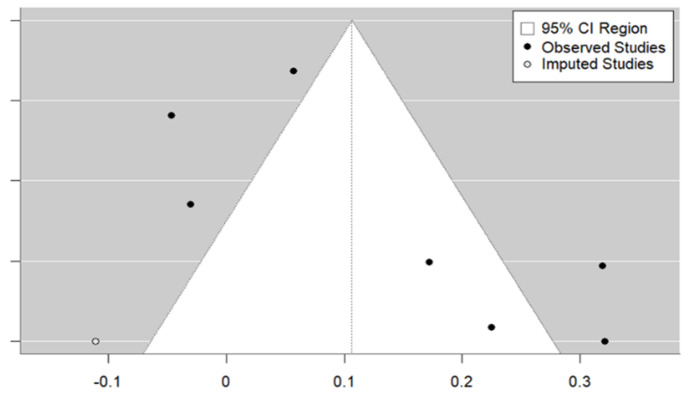
Writing strategy funnel plot.

Still on the subject of the writing strategy and according to the funnel plot (Figure 8), we can see that the sample is not representative, given the lack of symmetry, which is confirmed by Egger’s test (*p* = 0.0430). The trim and fill method identified one missing study. The overall estimate slightly decreases to 1.12 (95% CI: 0.99–1.25) when that missing study is included.

#### 3.3.5. Comparison of Strategies

Table 3 compares the type of intervention used to increase vaccination coverage with participant characteristics. Studies involving educational sessions demonstrated moderate heterogeneity $(I^{2}=59.4$) and proved to be the most effective strategy (OR=1.63, 95% CI: 1.22–2.19). Interventions conducted in countries with a high HDI were more effective (OR = 4.43, 95% CI: 2.06–9.53, $I^{2}=97.2$) than those in countries with a very high HDI (OR = 1.22, 95% CI: 1.07–1.39, $I^{2}=92.3$). However, it is important to note that all educational interventions were carried out in countries with a high HDI. Studies involving populations aged 60 or older (OR=4.92, 95% CI: 2.11–11.50, $I^{2}=97.5$) yielded better results than those involving populations aged 65 or older (OR = 1.26, 95% CI: 1.10–1.45, $I^{2}=92.3$).

## 4. Discussion

Vaccine hesitancy among individuals aged 60 and above represents a significant public health concern, as it serves as a contributing factor to the emergence of disease outbreaks and impedes the efficacy of epidemic control measures. One of the causes of this reluctance is the dissemination of misinformation among this population. It is, therefore, necessary to identify the most effective strategies for combating this misinformation [38]. Even among the elderly, interventions had varying effects, with younger elderly individuals achieving higher vaccination rates, consistent with previous studies [39]. The effect of the HDI of the country where the study was conducted was unclear, as all studies with educational strategies were conducted in countries with a high HDI, and no studies were retrieved from countries with low or medium HDIs.

A substantial corpus of literature exists that analyses the effectiveness of the various intervention strategies that have been employed in order to promote vaccination. Nevertheless, the existing literature on the efficacy of interventions in the elderly is limited. Additionally, numerous studies focus exclusively on the intention to vaccinate, rather than the actual vaccination rate, which can result in an inaccurate assessment of the intervention’s effectiveness [40,41,42]. With regard to the vaccines examined in these studies, influenza represents the most extensively studied vaccine in this age group. This is due to the fact that this age group is at a higher risk of developing complications, which consequently result in a higher incidence of morbidity and mortality on a global scale. Furthermore, as this demographic cohort advances in age, their immune system undergoes a decline in functionality [13]. It is, therefore, imperative to gain an understanding of the most efficacious strategies for reducing the number of complications and deaths worldwide.

An overall assessment of vaccination promotion strategies shows that they have a positive and significant impact (OR = 2.193, 95% CI; 1.431–3.361), but the high degree of heterogeneity ($I^{2}:99.50\%$) means that the interventions cannot be analysed as a homogenous group with similar effects. For instance, a study on the impact of free vaccination [23] found an adjusted OR of 30.46 (95% CI 20.86–44.49), which is clearly higher than almost all the other studies.

The implementation of educational strategies, including the distribution of information leaflets, the display of posters, the presentation of videos, the broadcasting of television programmes, and the provision of community counselling, has resulted in a notable and substantial increase in the vaccination rate among this age group. This increase can be attributed to the distribution of pamphlets in the waiting areas of health centres, clinics, and hospitals, prompting individuals to proactively seek out the material. Additionally, health professionals frequently provide pamphlets on the subject for later reference when recommending vaccination to their patients [22,33]. A previous review on vaccine hesitancy emphasized the importance of developing easy-to-read materials [43]. The increased urbanization of society has resulted in a significant shift in the location of medical practitioners, with many now working in urban health units. This has led to a lack of opportunity for these professionals to disseminate information regarding the importance of vaccination. Consequently, older individuals residing in urban areas have been primarily informed about this topic through television programmes, videos, and posts on community boards, with subsequent encouragement to accept vaccination [15,36,44].

Medical advice, including individualised advice and medical recommendations, has not been shown to have a positive and significant effect on increasing vaccination in the elderly, although it showed non-significant differences with education sessions. The observed high degree of heterogeneity between studies, as well as the low number of studies, limits the quality of the evidence and underlines the need for more studies. The relationship between doctor and patient has been identified as a factor influencing the decision to be vaccinated [35]. Regular consultations with a doctor have been found to positively influence vaccination rates, in comparison to those who do not have such regular contact with their doctors. In rural areas, medical professionals are more readily heeded due to the proximity of the population to healthcare services. This proximity facilitates the promotion of health and the implementation of medical recommendations [15].

The analysis of free and subsidised vaccination was not possible due to the very small number of studies (three) in this age group. However, given the high odds ratios reported, further studies are needed to confirm these results and to better understand their implications. This is a strategy that only emerged following the onset of the global pandemic caused by the SARS-CoV-2 virus, as there was a pressing need to eradicate the virus on a worldwide scale. The most effective method for reaching the largest number of people was to make vaccination mandatory. In fact, the studies found in this review were conducted after the emergence of COVID-19 [22,23,24].

Nevertheless, the extant studies indicate that greater accessibility to cost-free vaccination is a pivotal factor in enhancing the vaccination rate within this demographic. One effective strategy for augmenting this is the elimination of user fees for the elderly who are economically disadvantaged [15]. Additionally, it was discovered that a considerable number of individuals made voluntary donations with the intention of facilitating vaccine access for those with lower incomes. This approach has the potential to expand the reach of the vaccine to a larger population, which could be a significant contribution to social innovation in instances where government funding is unavailable [24].

The study revealed that written communication strategies, including letters, emails, and SMS messages, did not have a positive impact on vaccination rates among the elderly. This finding was attributed to technological and communication-related challenges, regardless of the brevity and personalisation of the messages [13,25]. One of the reasons, in the case of emails, is that a considerable number of messages were not read, which has an impact on the success or otherwise of the strategy. This is because it only works if the messages have been delivered and read [27,29]. Nevertheless, there is evidence to suggest that this strategy is indeed effective. The contacts provided for health records are often close family members who have greater access to new technologies [25].

The analysis revealed certain limitations inherent to the studies. The inclusion of only literate participants may have restricted the generalisability of the studies [33]. The restricted time for consultations and the scarcity of health professionals, particularly in the public sector, may have impeded the implementation of educational strategies, despite their favourable impact on the population [34]. The discrepancy between urban and rural areas may have influenced the acceptance of vaccination, as the methods employed are different, as previously described [15]. A study on vaccination coverage among the elderly in Poland suggests that rural areas have lower vaccination rates [39]. It is possible that the focus on the development and rollout of the SARS-CoV-2 vaccines may have indirectly contributed to a reduction in the uptake of other vaccines, such as those targeting influenza and pneumonia. It is plausible that these types of interventions could have been more effective if there had not been such a media spotlight [27]. Many of the included studies had issues identified in their quality assessments. Removing these from the analysis of each strategy would reduce the number of available studies to a very low level, compromising the conclusions of the sensitivity analysis. Therefore, this was not performed.

Finally, it would be beneficial for future studies to evaluate the effectiveness of combining strategies.

## 5. Conclusions

It is of the utmost importance to further our understanding of immunosenescence and to develop safer and more efficacious vaccines for this age group [13]. The efficacy of vaccination promotion strategies for this population varies. While it is not feasible to combine all strategies, the educational intervention, comprising information leaflets, posters, videos, and promotional activities on television and through community councils, demonstrated the greatest effectiveness. The recommendation of doctors has been identified as a key method for increasing vaccination rates, which are often associated with the distribution of informational leaflets. However, further research is required to elucidate the most effective strategies for enhancing vaccination uptake in this age group. This necessity emerges from investigations into uncosted vaccination and also into the significance of adherence to the HZ vaccine, given the dearth of studies on this subject for this age group.

The pharmacy has an important role to play in the promotion of immunization, a role that is often overlooked. In addition to administering vaccines, pharmacists are well positioned to assess patients’ histories and identify those at risk who require immunization. Furthermore, they can encourage vaccination [37]. The number of studies in this context is still very limited, and those that do exist do not cover this age group. Further investigation is required to ascertain whether this subject has a positive effect or not. However, while further research is needed, it is evident that the choice of strategy is significant.

## Figures and Tables

**Table 1:** Summary of the studies included for analysis. (HDI: Human Development Index 2024, OR: odds ratio, NA: not available, * adjusted OR).

Study	Country	HDI	Rural/ Urban	Age Mean	Female (%)	Type of Study	Vaccine	Population	Sample Size	Type of Strategy	OR (95% CI)
Abu-rish & Barakat, 2021 [28]	Jordan	High (0.736)	Urban	70.0	78.8	Intervention study	Pneumococcal	Unvaccinated ≥ 65 years old	700	Educational charter	* 1.95 (1.35–2.81)
Du et al., 2023 [22]	China	High (0.788)	Rural and urban	NA	NA	Quasi-experimental trial	*Influenza*	Unvaccinated ≥ 60 years old	225	Free and subsidised vaccination	6.91 (3.59–13.32)
Esteban-Vasallo et al., 2019 [25]	Spain	Very high (0.911)	Mostly urban	NA	NA	Quasi-experimental study, pre/post intervention	*Influenza*	Unvaccinated > 64 years old with concomitant comorbidities and rare disease	17695	SMS	1.38 (1.21–1.57)
Ho et al., 2019 [35]	Singapore	Very high (0.949)	Urban	NA	55.6	Randomised, crossover study	*Influenza*	Unvaccinated people aged ≥ 65 years, with or without illness and who have visited the clinic	4378	Information brochures	* 1.43 (0.99-2.07)
							Pneumococcal				* 1.78 (1.28-2.48)
Hu et al., 2023 [33]	Singapore	Very high (0.949)	Urban	71.0	49.1	Randomised clinical trial	*Influenza*	Unvaccinated ≥ 65 years old	320	Individualised counselling	* 1.29 (0.64–2.58)
Jiang et al., 2021 [23]	China	High (0.788)	NA	69.5	47.2	Quasi-experimental trial	*Influenza*	Unvaccinated people aged ≥ 60 from local health centres	1210	Free vaccination	* 30.46 (20.86–44.49)
Jiang et al., 2022 [36]	China	High (0.788)	Urban	NA	54.9	Randomised clinical trial	*Influenza*	Unvaccinated ≥ 60 years old	350	Educational video	* 3.90 (1.41–10.75)
Johansen et al., 2023 [31]	Denmark	Very high (0.952)	NA	NA	NA	Randomised clinical trial	*Influenza*	Unvaccinated ≥ 65 years old with heart failure, excludes residents of nursing homes	384913	Electronic letters	1.06 (1.03–1.09)
Leung et al., 2017 [29]	Hong Kong	Very high (0.956)	Urban	74.6	52.5	Randomised clinical trial	*Influenza*	Unvaccinated ≥ 65 years old	529	Individualised verbal education	1.62 (1.11–2.35)
Lieu et al., 2022 [37]	United States	Very high (0.927)	NA	72.6	56.3	Randomised clinical trial	COVID-19	Unvaccinated ≥ 65 years old	3858	Electronic messages	1.25 (1.06–1.47)
										Letters	1.19 (1.04–1.35)
Lv et al., 2016 [15]	China	High (0.788)	Mostly urban (83.2%)	NA	57.2	Retrospective cross-sectional study	*Influenza*	Unvaccinated people who have lived in Beijing for at least one year and are ≥ 60 years old	1673	Community counselling	* 1.812 (1.446–2.27)
										Television	* 1.18 (0.97–1.45)
										Doctors’ recommendations	* 3.31 (2.64–4.15)
Regan et al., 2017 [26]	Australia	Very high (0.946)	Mostly urban	NA	NA	Randomised clinical trial	*Influenza*	Unvaccinated ≥ 65 years old	3613	SMS reminders	* 1.38 (1.16–1.63)
Sääksvuori et al., 2022 [30]	Finland	Very high (0.942)	Rural	75.5	NA	Randomised clinical trial	*Influenza*	Unvaccinated ≥ 65 years old with no vaccination data from the previous year	7324	Letter reminders	0.97 (0.88–1.07)
Szilagyi et al., 2023 [27]	United States	Very high (0.927)	Mostly urban	NA	NA	Randomised clinical trial	*Influenza*	Unvaccinated ≥ 65 years old	39235	SMS reminders	0.95 (0.91–1.00)
Wu et al., 2022 [24]	China	High (0.788)	Rural (33%) and urban (67%)	68.0	68.7	Quasi-experimental trial	*Influenza*	Unvaccinated ≥ 60 years old	150	Subsidised vaccine	* 5 (2.3–10.08)
You et al., 2023 [32]	China	High (0.788)	Urban	NA	NA	Experimental study	*Influenza*	Unvaccinated ≥ 60 years old	2158	Doctors’ recommendations	12.93 (9.33–17.93)

**Table 2:** Quality of observational studies (Newcastle–Ottawa Scale). Each star represents a point on the scale.

Study ID	Items								
	Selection				Comparability	Exposure			Score
	1	2	3	4	1	1	2	3	
Abu-rish & Barakat, 2021 [28]	*	*	-	*	**	-	*	*	7/9
Du et al., 2023 [22]	*	*	*	-	**	-	*	-	6/9
Esteban-Vasallo et al., 2019 [25]	*	*	*	*	**	*	*	*	9/9
Ho et al., 2019 [35]	*	*	-	*	**	*	*	*	8/9
Jiang et al., 2021 [23]	*	*	*	-	**	-	*	*	7/9
Lv et al., 2016 [15]	*	*	*	-	**	-	*	*	7/9
Wu et al., 2022 [24]	*	*	*	*	**	*	*	*	9/9
You et al., 2023 [32]	*	*	-	-	**	*	*	*	8/9

**Table 3:** Comparison of strategies. (N.s: number of studies, N.i: number of individuals, OR: odds ratio, HDI: Human Development Index, *p*-values lower than 0.05 are highlighted in bold).

Subgroup	N.s (N.i)	OR (95% CI)	$I^{2}$	*p*-Value
Type of intervention				**0.019**
Educational	6 (4378)	1.63 (1.22–2.19)	59.4	
Medical advice	4 (2158)	3.13 (0.60–16.37)	97.0	
Writing	7 (384,913)	1.14 (0.99–1.32)	93.3	
HDI				**<0.001**
High	9 (2158)	4.43 (2.06–9.53)	97.3	
Very high	11 (384,913)	1.22 (1.07–1.39)	92.3	
Age (mean)				0.099
≤71	4 (1210)	4.43 (0.54–36.6)	96.6	
>71	4 (7324)	1.18 (0.88–1.58)	79.3	
Age (minimum)				**<0.001**
60	8 (2158)	4.92 (2.11–11.50)	97.5	
65	12 (39,235)	1.26 (1.10–1.45)	93.4	
Female (%)				0.935
≤57	8 (3858)	2.27 (0.95–5.41)	98.2	
>57	5 (1673)	2.29 (1.10–4.78)	93.7	

## Data Availability

The datasets used and/or analysed during the current study are available from the corresponding author upon reasonable request.

## Supplementary Materials

The following supporting information can be downloaded at https://www.mdpi.com/article/10.3390/vaccines12121395/s1, Table S1: Newcastle–Ottawa Quality Assessment Scale for case–control studies (adapted).
