# Supplementary material for: Vaccination Promotion Strategies in the Elderly: Systematic Review and Meta-Analysis
Source: Vaccines (Basel). 2024 Dec 11;12(12):1395. doi: 10.3390/vaccines12121395 (PMC11728613; doi:10.3390/vaccines12121395)
Supplement: Supplementary file 1 [file vaccines-12-01395-s001.zip › vaccines-3350206-supplementary.pdf]

Supplementary Table S1. Newcastle - Ottawa Quality Assessment Scale for case control studies (adapted)

NEWCASTLE - OTTAWA QUALITY ASSESSMENT SCALE
CASE CONTROL STUDIES

Note: A study can be awarded a maximum of one star for each numbered item within the Selection and Exposure categories. A maximum of two stars can be given for Comparability.

Selection

1) Is the case definition adequate?
   a) yes, with independent validation ❋
   b) yes, eg record linkage or based on self reports
   c) no description

2) Representativeness of the cases
   a) consecutive or obviously representative series of cases ❋
   b) potential for selection biases or not stated

3) Selection of Controls
   a) community controls ❋
   b) hospital controls
   c) no description

4) Definition of Controls
   a) no prior vaccination ❋
   b) no description of source

Comparability

1) Comparability of cases and controls on the basis of the design or analysis
   a) study controls for gender ❋
   b) study controls for any additional factor ❋

Exposure

1) Ascertainment of exposure
   a) secure record (eg medical record) ❋
   b) self report
   c) no description

2) Same method of ascertainment for cases and controls
   a) yes ❋
   b) no

3) Non-Response rate
   a) same rate for both groups ❋
   b) non respondents described
   c) rate different and no designation
